# A frailty census of older adults in the emergency department and acute inpatient settings of a model 4 hospital in the Mid-West of Ireland

**DOI:** 10.1007/s11845-024-03775-6

**Published:** 2024-09-19

**Authors:** Ida Carroll, Aoife Leahy, Margaret O.’ Connor, Nora Cunningham, Gillian Corey, David Delaney, Sheila Ryan, Aoife Whiston, Rose Galvin, Louise Barry

**Affiliations:** 1 Ageing Research Centre, Limerick, Ireland; 2 School of Allied Health, University of Limerick, Limerick, Ireland; 3 Department of Nursing and Midwifery, University of Limerick, Limerick, Ireland; 4 University Hospital Limerick, Dooradoyle, Co Limerick Ireland; 5 Department of Ageing and Therapeutics, Limerick, Ireland; 6 Local Injury Unit, Ennis General Hospital, Ennis, Ireland; 7 Thurles Ambulatory Care Hub for Older Persons, Thurles, Ireland

**Keywords:** Charlson Co-morbidity Index, Clinical Frailty Scale, Frailty screening, Older Person Services, Point of prevalence

## Abstract

**Background:**

Frailty is a risk factor for presentation to the ED, in-hospital mortality, prolonged hospital stays and functional decline at discharge. Profiling the prevalence and level of frailty within the acute hospital setting is vital to ensure evidence-based practice and service development within the construct of frailty. The aim of this cross-sectional study was to establish the prevalence of frailty and co-morbidities among older adults in an acute hospital setting.

**Methods:**

Data collection was undertaken by clinical research nurses and advanced nurse practitioners experienced in assessing older adults. All patients aged ≥ 65 years and admitted to a medical or surgical inpatient setting between 08:00 and 20:00 and who attended the ED over a 24-h period were screened using validated frailty and co-morbidity scales. Age and gender demographics, Clinical Frailty Scale (CFS), Charlson Co-morbidity Index (CCI) and admitting specialty (medical/surgical) were collected. Descriptive statistics were used to profile the cohort, and *p* values were calculated to ascertain the significance of results.

**Results:**

Within a sample of 413 inpatients, 291 (70%) were ≥ 65 years and therefore were included in the study. 202 of these 291 older adults (70%) were ≥ 75 years. Frailty was investigated using validated clinical cut-offs on the CFS (not frail < 5; frail ≥ 5). Comorbidities were investigated using the Charlson Comorbidity Index (mild 1–2; moderate 3–4; severe ≥ 5). The median CFS was 6 indicating moderate frailty levels, and the median CCI score was 3 denoting moderate co-morbidity. In the inpatient cohort, 245 (84%) screened positive for frailty, while 223 (75%) had moderate-severe co-morbidity (CCI Mod 3–4, severe ≥ 5). No significant differences were observed across genders for CFS and CCI. In the ED, 81 patients who attended the ED were ≥ 65 years. The median CFS was 6 (moderate frailty), and the median CCI was 5 (severe co-morbidity level). Seventy-four percent (60) of participants screened positively for frailty (CFS ≥ 5), and 31% (25) had a CFS of 7 or greater (severely frail). Ninety-six percent (78) of patients had a moderate-severe level of comorbidity. No significant associations were found between the CFS and CCI and ED participants age, gender, and medical/surgical speciality usage.

**Conclusion:**

There is a high prevalence of frailty and co-morbidity among older adults who present to the ED and require inpatient care. This may contribute to increased waiting times, lengths of stay, and the need for specialist intervention. With an increased focus on the integration of care for older adults across care transitions, there is a clear need for expansion of frailty-based services, staff training in frailty care and multidisciplinary team resources across the hospital and community setting.

**Supplementary information:**

The online version contains supplementary material available at 10.1007/s11845-024-03775-6.

## Background

Frailty is a contemporary construct which is increasingly recognised as a distinctive state of health, related to the aging process, in which multiple body systems gradually lose their inbuilt reserves [1]. Frailty is not an inevitable part of ageing; it is a long-term condition in the same sense as diabetes or Alzheimer’s disease [2]. Frailty is usually described in terms of frailty phenotype or frailty index [3]. The frailty phenotype looks for a predetermined set of criteria such as weight loss, exhaustion, slow gait speed, poor hand grip strength, sedentary behaviour, falls, immobility, delirium, incontinence or the effects of polypharmacy [3–7]. The frailty index views frailty as a dynamic state, describing it as an accumulation of deficits and takes a fluid approach to frailty assessment. However, validated frailty screening tools have consistently shown to be reliable in pin-pointing those at risk of adverse outcomes.

The assessment and management of frailty are an emerging and developing concept, aiming to identify vulnerable older patients and anticipating their unique healthcare needs [1]. Early identification and systematic assessment and management are recognised as an optimal approach in older person care. Frailty is a risk factor for in-hospital mortality, prolonged hospital admission and functional decline at discharge [8]. Profiling the prevalence and level of frailty within the acute hospital setting is vital to ensure evidence-based practice and service development within the construct of frailty [9]. Identifying the most at-risk older people in EDs may help guide service improvement and clinical practice in emergency care [10]. Older adults, people aged 65 and over, comprise 12.7% of the Irish population and use 53% of inpatient beds [11]. In addition, 25% of all attendees to the ED are older and experience complex co-morbidities and healthcare needs [12]. According to Robinson and Galvin [13, 30], an opportunity exists to provide timely specialist assessment and intervention to some older adults seeking emergency care with a view to reducing the risk of long ED waiting times and avoidable hospital admissions. This is particularly significant in the Mid-West of Ireland where ED waiting times are chronically prolonged and older adults can avail of alternative pathways to acute services if appropriate. Furthermore, according to Cummins et al. [14], in the mid-west of Ireland, challenges with flow and capacity in acute hospital settings and community services highlight that solutions to ED crowding may lie largely outside of the ED. If capacity is not provided, along with sustainable system-wide solutions aimed at managing complex patient care provision, ED crowding will remain a significant public health issue in the mid-west [14]. Considering the ageing population in Ireland, the increasing incidence of frailty and comorbidity with age, and the continued reliance of older adults on acute services, monitoring the prevalence and level of frailty and comorbidity is key to inform service planning and development and referral to alternative out of hospital pathways. The aim of this cross-sectional study was to measure the prevalence of frailty and co-morbidities among older adults in an acute hospital setting (CCI and CFS Supplementary File One). A secondary aim was to explore the association between comorbidity and frailty and participants’ age, gender, and admitting specialty.

## Methodology

### Study design

This represents a cross-sectional study to capture the prevalence of frailty and comorbidities among older adults in an acute hospital setting over a 12-h period (08:00–20:00) and in the Emergency Department (ED) over a 24-h period (12 pm on Monday to 11:59 am on Tuesday). The STROBE standardised reporting guidelines were followed to ensure the standardised conduct and reporting of the study [15] (Supplementary File Two).

*Ethical approval*

Ethical approval was granted for both the inpatient and ED census by the local research ethics committee (Research Ethics Committee Approval Number 122/2021).

### Selection/recruitment

All patients over 65 who were admitted to a medical or surgical inpatient ward during the 12-h period on the census day were suitable for inclusion. A 12-h period was chosen to measure inpatient frailty as it represents the peak hours for patient admission. Any current inpatients over 65 on these wards were also suitable for inclusion. Excluded patients included those admitted to the department of psychiatry and those in the critical care block. This cohort was excluded as many of these patients were acutely ill and, consequently, had high levels of frailty associated with their clinical presentation.

In the ED census, all patients over 65 who attended the ED or were subsequently seen in an acute assessment unit (Medical Assessment Unit (MAU)/Surgical Assessment Unit (SAU)/Short Stay Assessment Unit (SSAU)) were suitable for inclusion. This pathway through the ED, triage to relevant acute assessment units, has been undertaken since the onset of the COVID-19 pandemic. The number of older adults presenting to the ED is relatively consistent; therefore, it was deemed appropriate that a 24-h data collection would reflect the level of frailty among this cohort.

### Variables of interest

For the purposes of this study, frailty was measured using the Rockwood Clinical Frailty Scale (CFS). This CFS has been validated and widely used in multiple clinical settings and is highly predictive of adverse outcomes among individuals over the age of 65 [16, 31]. This is a user-friendly tool, which can be quickly applied by knowledgeable staff to objectively rate frailty in older adults. The scale requires clinicians to consider patients’ physical, functional, psychological, and social ability. The Charlson Comorbidity Index (CCI) is a validated, simple, and readily applicable method of estimating risk of death from comorbid disease and is widely used as a predictor of long-term prognosis and survival, and it is a summary measure to give clinicians and researchers a single number that captures this information [17]. According to Charlson et al. [18], the CCI can be clinically useful not only to provide a valid assessment of the patient’s unique clinical situation but also to demarcate major diagnostic and prognostic differences among subgroups of patients sharing the same medical diagnosis. Importantly, the clinimetric sensitivity of the CCI has also been demonstrated in a variety of medical conditions, with stepwise increases in the CCI associated with stepwise increases in mortality [29]. The CCI score will also be measured to give insight into clinical prognosis and diagnosis along with the patients’ level of frailty. Demographics including age and gender and medical/surgical speciality care provision were also noted to inform crosstabulation of results for significance in terms of the prevalence and severity of frailty and co-morbidity.

### Data collection

Data collection was undertaken by clinical research nurses and advanced nurse practitioners who work with older adults routinely, were familiar with the hospital setting and who routinely employ frailty screening as part of their role. Relevant scales and demographic information were collected using data from the patients’ medical notes. Only numerical values and the patients’ gender and medical/surgical presentation were recorded. These values were noted under an anonymous participant number.

### Data analysis

Descriptive statistics were calculated for all included variables. This included frequency statistics for categorical variables and means (standard deviations) for scale variables. For each cohort—ED and inpatients, chi-square tests of association were conducted to see if distributions of frailty and comorbidities significantly differed across age (< 75 and ≥ 75), gender (male and female), medical admission (yes vs. no), and surgical admission (yes vs. no). Frailty was investigated using validated clinical cut-offs on the CFS (not frail < 5; frail ≥ 5). Comorbidities were investigated using the Charlson Comorbidity Index (mild 1–2; moderate 3–4; severe ≥ 5).

## Results

Data were collected on 291 inpatients and 81 older adults attending the ED. Please see Supplementary File Three for Methods and Analysis Breakdown.

### Inpatient cohort results

Out of a possible 413 adult inpatients, 291 were over the age of 65. Therefore, 70% of inpatients were over the age of 65 at the time of the study and suitable for inclusion. Fifty-four percent were male, and 65% were over 75. Eighty-four percent of those over 65 screened positive for frailty as per the CFS. Forty-five percent (132) had a moderate level of comorbidity with 31% (91) rated at a severe level of comorbidity on the CCI. Thirty-seven patients were under the joint care of medicine and surgery. Seventy-one percent were either admitted or consulted by medical teams. The following tables present an overview of cohort demographics and census results and a breakdown of the CFS measurements (Tables 1, 2, and 3). Median CFS 6 (moderate), Median CCI 3 (moderate). The mean and standard deviations for each scale are represented below.

**Table 1:** Inpatient cohort demographics and summative presentation of results

**Demographic**	**Number**	**Percent**
** < 75**	104	35.7%
** ≥ 75**	187	64.3%
**Male**	158	54.3%
**Female**	133	45.7%
**Admitting speciality medical: no**	84	28.9%
**Admitting speciality medical: yes**	207	71.1%
**Admitting speciality surgical: no**	170	58.4%
**Admitting speciality surgical: yes**	121	41.6%
**CFS: not frail**	46	15.8%
**CFS: frail**	245	84.2%
**CCI: mild**	68	23.4%
**CCI: moderate**	132	45.4%
**CCI: severe**	91	31.3%

**Table 2:** An overview of cohort demographics and census results and a breakdown

	***N***	**Range**	**Minimum**	**Maximum**	**Mean**	**Std. deviation**
**CFS score**	291	6	2	8	5.83	1.275
**CCI score**	291	11	1	12	4.04	2.342
**Valid** ***N*** **(listwise)**	291

**Table 3:**
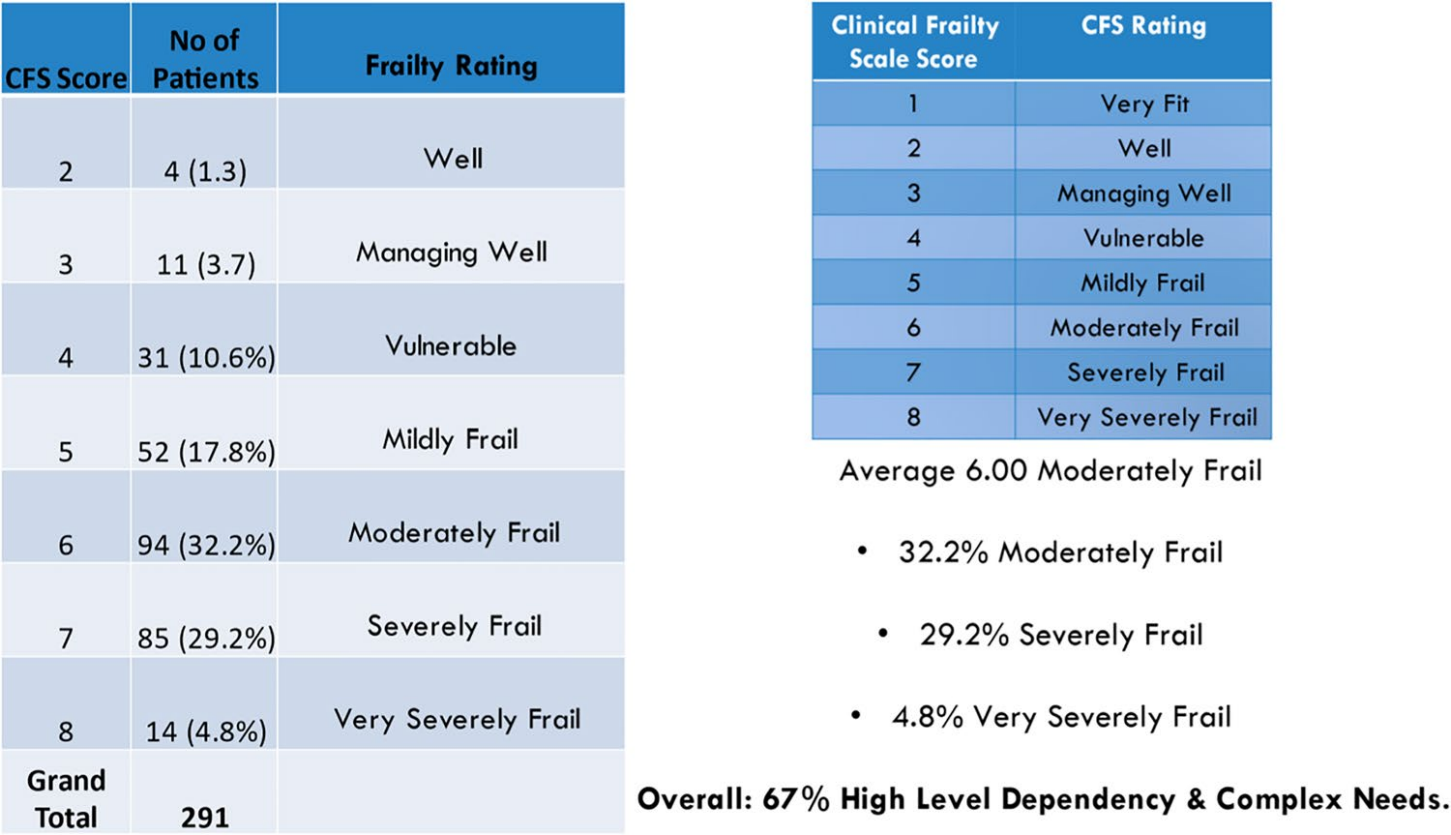
Rates of inpatient frailty as per CFS

### CFS inpatient analysis chi-squared tests of association

The relevant results pertaining to the CFS relationship with age, medical vs. surgical admission, and gender were cross tabulated. Those ≥ 75 were more likely to screen as frail (*p* = 0.002). There was no association found between CFS frailty and gender (*p* = 0.144). Medical admissions were more likely to be frail (*p* = 0.004). In this instance, the level of frailty among those admitted to the surgical speciality was still significant (84%). A higher % of surgical patients were not frail; however, there was still a significant relationship between surgical speciality usage and the prevalence of those screening as frail (*p* = 0.006).

### CCI inpatient observations

Overall, 75% of those measured had moderate to severe levels of comorbidity. Tables 4 and 5 below give a breakdown of the level of comorbidity among the inpatient cohort.

**Table 4:**
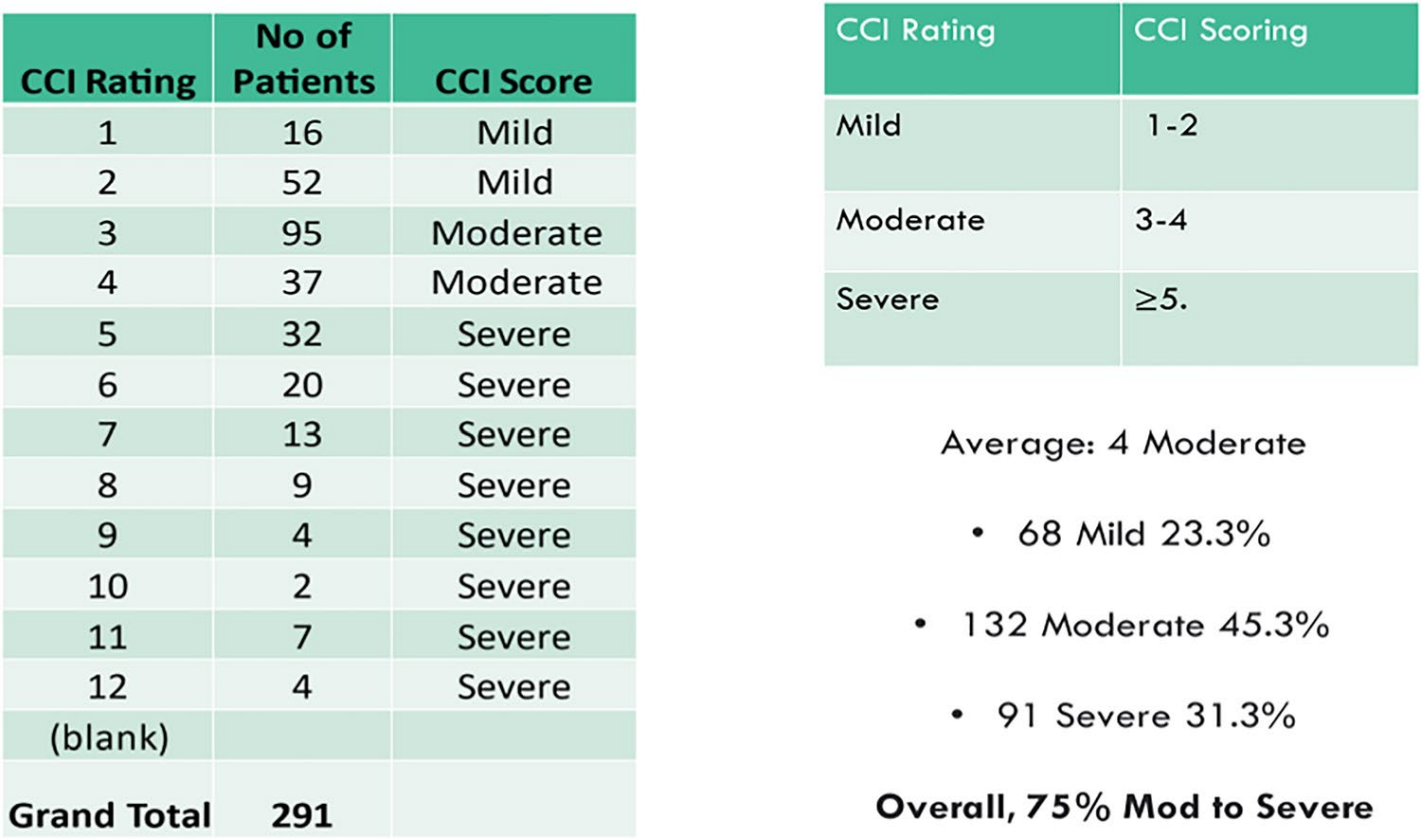
Rates of inpatient co-morbidity

**Table 5:** Mean CFS and CCI by age

**Age range**	**No. in range**	**Clinical Frailty Scale Average**	**Charlson Comorbidity Index Average**
65–70	43	5.11 Mildly frail	3.7 Moderate
70–75	61	5.52 Mildly frail	3.8 Moderate
75–80	68	6.04 Moderately frail	4.2 Moderate
80–85	70	6.00 Moderately frail	4.08 Moderate
85–90	32	6.12 Moderately frail	3.06 Moderate
90–95	14	6.71 Moderately frail	4.2 Moderate
95–100	3	6.66 Moderately frail	3.3 Moderate

### CCI analysis chi-squared tests of association

The CCI relationship with age, medical vs. surgical admission, and gender were cross-tabulated. Increasing age was associated with more severe rates of comorbidity (*p* = 0.026). No association was found between medical admission and severity of co-morbidity (*p* = 0.227). Similarly, no association between surgical admission and severity of frailty was found (*p* = 0.620). No association between gender and severity of co-morbidity was detected (*p* = 0.465). The predominant relationship was observed between age and frailty and CCI severity. Table 5 illustrates the progression of frailty and comorbidity associated with ageing.

### ED cohort

In the cohort reviewed in the ED (Table 6), 57% of patients were female and 63% were over the age of 75. With increasing age, the average CFS score increased with those over > 75 being moderately frail. The median CFS was 6 (moderate), and the median CCI was 5 (severe). Overall, 74%, 60/81 patients, screened as frail. Furthermore, 96% of patients had moderate to severe co-morbidities. The distribution and rates of CFS in the ED are outlined in Table 7. The mean and standard deviations for each scale are represented below under descriptive statistics (Table 8).

**Table 6:** ED cohort demographics and summative presentation of results

**81 patients**		
**Demographic**	**Number**	**%**
< 75	30	37.0%
≥ 75	51	63.0%
Male	35	43.2%
Female	46	56.8%
Speciality medical: no	18	22.2%
Speciality medical: yes	63	77.8%
Speciality surgical: no	53	65.4%
Speciality surgical: yes	28	34.6%
CFS: not frail	21	25.9%
CFS: frail	60	74.1%
CCI: mild	3	3.7%
CCI: moderate	27	33.3%
CCI: severe	51	63.0%

Note some patients Surgical and Medical Care

**Table 7:** Distribution and rates of clinical frailty in the ED

**CFS score**	**No. of patients**	**%**
2 Well	2	2.5%
3 Managing well	6	7.4%
4 Vulnerable	13	16.0%
5 Mildly frail	15	18.5%
6 Moderately frail	20	24.7%
7 Severely frail	20	24.7%
8 Very severely frail	5	6.2%

**Table 8:** Descriptive statistics

	***N***	**Range**	**Minimum**	**Maximum**	**Mean**	**Std. deviation**
**CFS_SCORE**	**81**	**6**	**2**	**8**	**5.54**	**1.484**
**Charlson_SCORE**	**81**	**12**	**1**	**13**	**5.75**	**2.672**
**Valid** ***N*** **(listwise)**	**81**					

### CFS ED observations

In contrast to the inpatient cohort, no clear association was found between age and frailty among those screened in the ED (*p* = 0.153). No association between medical specialty usage and frailty was found (*p* = 0.084). Furthermore, no association between surgical admissions and frailty was found (*p* = 0.232). In congruence with the inpatient cohort, no association between gender and frailty was found (*p* = 0.465).

### CCI ED observations

Cross tabulations for the CCI were run on the moderate and severe groups only due to the mild group having insufficient counts to be included in the analysis (*n* = 3). No association was found between CCI and age range (*p* = 0.252). No associated was found between the CCI and medical speciality usage (*p* = 0.147), surgical speciality usage (0.614), and gender (*p* = 0.396).

Overall, the level of frailty and co-morbidity across the ED patient cohort was high. However, upon cross-tabulation of the significance of results across age, gender, and medical/surgical speciality, no statistically significant associations were found.

## Discussion

This cross-sectional study identified a high prevalence of frailty and co-morbidity among older adults attending the ED and in acute inpatient settings. In Ireland, the rate of physical frailty consistently increases with age [1]. Similarly, within the inpatient sample group, the rate of screening positive for frailty increased with age and the overall rates of older adults with a moderate to severe level of comorbidity were high in both cohorts. However, this higher incidence is not surprising with the TILDA [1] report identifying frailty among Over 55 as a proportion of the total population of Ireland, as having a higher prevalence in the mid-west of Ireland (Limerick 0.9%) and among those 70 + (Limerick 1.2%). Considering the limited resources across the Irish health service, keeping abreast of patients’ complex healthcare requirements is becoming more and more challenging. This study highlights the degree of complexity and frailty in this cohort and mirrors the frailty prevalence in a similar cohort of ED patients where 57–70% of patients screened positive for frailty (the variation related to the screening tool used) [19]. Several studies [10, 19] have shown the adverse outcomes associated with screening positive for frailty in older adults who present to ED. They have been shown to have increased risk of mortality, ED representation, hospitalisation, functional decline, and nursing home admission [19]. Therefore, acute intervention with this vulnerable group is vital to ensure consistent reduction in adverse outcomes.

Of the 80,600 adults aged 70 + years living with frailty, 44,500 (55.2%) do not receive any form of informal care or formal community support, 24,800 (30.8%) receive informal care from a family member or friend, 26,100 (32.4%) receive formal community support services, and 7600 (9.4%) pay for private home help or a personal care attendant [1]. Although there is a clear awareness of the prevalence of frailty in Ireland and the mid-west, the severity and functional incapacity of this cohort is unknown. As reflected, many of these older adults require additional supports and intervention. The levels of frailty are high in the mid-west of Ireland, and this is reflected in this snapshot of frailty in acute settings. This is a concern considering that healthcare services across the mid-west are already facing challenges in meeting the needs of a complex and ageing population. Furthermore, although women are more likely to be frail [20], this study has not found any association between screening positive for frailty/co-morbidity and gender. The rates of frailty co-morbidity were found to be consistent across age groups and genders. Therefore, the importance of an overarching approach to screening and intervention is further emphasised.

A recent systematic review and meta-analysis by Boucher et al. (2023) [9] highlighted variation in the prevalence of frailty across different studies and different assessment tools. However, this review emphasised that all patients had an increased risk of adverse outcomes if they screened positive for frailty. Furthermore, this review highlighted that those who were frail were more likely to have increased mortality, increased length of stay, and more severe frailty upon discharge. As the body of evidence grows in relation to this, it is of utmost importance to identify those who are frail during their inpatient stay [9]. The evident presentation of mod-severe co-morbidities among this patient cohort further compounds the need for CGA (Comprehensive Geriatric Assessment) in the ED to reduce the risk of adverse outcomes in high-risk patients.

Furthermore, it has been recommended by international geriatric medicine forums that frailty screening is administered in the ED setting to identify those at risk [19]. However, this has not been reviewed at a hospital-wide level. Given the magnitude of those screening positive for frailty in our cohort, a system-wide approach to the identification of frailty in older adults across the entire acute hospital spectrum, including all specialities, is recommended. According to a systematic review and meta-analysis by Doody et al. (2022) [21], which looked at 96 studies with a pooled sample of 467,779 geriatric hospital inpatients, frailty is highly prevalent among geriatric hospital inpatients (50% deemed frail). This cohort of patients was heterogenous, and this was based on the analysis of clinical and demographic characteristics [21]. This review highlights the increase of frailty which has progressed throughout the healthcare system [21]. Considering the longer-term impact of an ageing population in Ireland and the increased prevalence of frailty, mapping frailty and co-morbidity prevalence across both the acute and community settings may help to predict forthcoming challenges and the need for service development. In addition, a systematic review by Rezaei-Shahsavarloo et al. (2020) [22] found that multidimensional interventions conducted by a multidisciplinary specialist team in geriatric inpatient settings are likely to be effective in the care of hospitalized frail older adults. This was encouraging, however, they highlighted that further evidence is needed to ascertain the value of various forms of holistic multidisciplinary care provision, and this included CGA and telemedicine programmes. Therefore, further evidence is required to underpin appropriate interventions for those who screen as frail or with co-morbidities as inpatients.

Evidently, the prevalence of frailty and co-morbidity, particularly among patients who present with acute medical complaints, is a concern. This further highlights the need for early identification and intervention among older adults who present to acute services. In Ireland, to meet the needs of older adults, models of acute geriatric care for patients presenting to the ED with acute medical complaints are becoming more common. These include acute geriatric units (AGU), frailty at the front door services (FFD), and numerous acute assessment units and teams who deliver care specifically to older patients. This acute care provision has been found to improve clinical and process outcomes for hospitalised older adults with acute medical complaints [23, 24]. It has also been recommended that clinicians utilise frailty criterion when selecting older adults for admission to AGUs [23]. Therefore, embedding frailty screening, within ED services, is vital to identify patients most suitable for assessment and must be reinforced. This can be particularly challenging in the ED setting, however, a deliberate approach to the implementation of screening in the ED which considers preconditions to facilitating screening, exploring staffs’ motivations to screen and instilling the knowledge and skills required to screen among clinicians can foster a screening culture within the ED [25].

This study has further illustrated that frailty occurs across both medical and surgical specialities. Therefore, a universal approach to screening and intervention, considering the needs of each speciality, is warranted. According to a systematic review by Lin et al. (2016) [26], there is robust evidence that frailty among older surgical patients predicts post-operative mortality, complications, and prolonged length of stay, and therefore, frailty assessment may be a valuable tool in peri-operative assessment. The perioperative multidisciplinary team believes that frailty assessment and management should play a role in patient care planning [27]. However, few reported screening in practice with many identifying barriers to engagement, and this included a lack of knowledge pertaining to the use of frailty assessments and a need for training [27]. The CCI is also a valuable tool to predict mortality and readmission among older surgical patients, particularly if age-adjusted [28]. This tool has potential to stratify high-risk older patients for surgical procedures [28]. Further emphasis and reinforcement of perioperative frailty screening and co-morbidity rating is warranted to ensure an overarching and standardised approach to the assessment of older adults. The association between frailty and return to pre-morbid function, discharge destination, and quality of life after surgery also warrants further research [26].

## Strengths and limitations

In terms of study strengths, the frailty screening and comorbidity rating were performed by nursing staff specialised in geriatric nursing and accustomed to administering these screening tools. This has enhanced the accuracy of frailty and comorbidity measurement. In addition, all eligible participants were surveyed as part of this study which required rigorous protocol development and planning on the part of an experienced research team. This study is limited as it is a snapshot of a single day but aligns with similar cohort studies in this population. The transparent reporting of the study process has enhanced the reproducibility and dependability of study findings. Difficulties exist when modelling complex temporal patterns that combine non-linear and seasonal patterns and are sensitive to the effects of national and local conditions [1, 32]. To further establish associations between patient demographics and frailty/comorbidity, particularly in the ED, further data collection and interrogation of frailty and co-morbidity measurements are warranted and will be undertaken.

## Conclusion

The incidence of older adults screening positive for frailty in both the emergency department and acute inpatient settings is concerningly high. Identifying this frail cohort of older adults is recommended to ensure that a comprehensive interdisciplinary assessment is employed, and resources are allocated in an informed and evidence-based manner. Considering the current challenges in delivering holistic interdisciplinary care to this cohort in both the acute and community setting, mapping frailty among the older cohort is pivotal to ensure a well-informed, purposeful, and efficient use of resources. PPI involvement is also vital to ensure that resource allocation is supported and informed by those who avail of older person specific healthcare services. In Ireland, the development of community services, older person specific acute services, and advanced and specialist nursing practice has emphasised the need to stay abreast of complex challenges experienced by older adults. However, to articulate the ever-evolving needs of this complex group, measuring frailty and co-morbidity incrementally can assist in identifying areas for development and expansion relating to both material and human resources. In Ireland, the ongoing rollout of the National Frailty Education Programme, the continuing progression of the National Clinical Programme for Older Adults, the Integrated care Framework for Older Adults with an emphasis on frailty screening and CGA and the development of geriatric specific acute units in Emergency Departments nationally is encouraging. Frailty at the front door and ANP led geriatric services are welcomed and promising. Furthermore, the development of specialist community integrated care hubs to meet the needs of this older patient cohort is crucial particularly with the moderate-severe level of comorbidity measured.

## Recommendations

Considering the fluctuating demand for acute services in the mid-west, with the Winter period consistently resulting in increased presentations, this census should be repeated quarterly to allow for seasonal changes in demand and to inform resource allocation. In addition, an acute inpatient frailty liaison service to intervene with CGA among complex moderate to severely frail patients may reduce the risk of adverse outcomes. Furthermore, the development of standards of care to underpin frailty services would ensure a consistent and evidence-based approach. To meet the needs of this complex patient cohort, the National Integrated Care Programme for Older People (NICPOP) and the National Clinical Programme for Older People (NCPOP) are leading out on the development of cohesive primary and secondary care services for older people especially those with more complex needs [11]. These programmes have changed the way in which care of the older person is organised and delivered in Ireland, with a focus on the development of an end-to-end pathway that provides cohesive primary (ICPOP Specialist Ambulatory Care Hubs), secondary (Rapid Response Specialist Care in the Community), and acute care services (Frailty at the Front Door (ED/AMAU)) for older people with a specific focus on those with more complex needs and frailty. The focus on identifying those who are at risk of adverse outcomes, who screen positive for frailty with comorbidities, is vital to this process and needs to be integrated consistently throughout the care pathway. Finally, reinforcing the value of frailty screening and CCI rating among perioperative multidisciplinary teams is required to provide comprehensive and overarching assessment and care to older adults who present to acute services in the mid-west.

## Supplementary information

Below is the link to the electronic supplementary material.

Supplementary file1 (PDF 238 KB)

Supplementary file2 (PDF 90 KB)

Supplementary file3 (PDF 479 KB)

## Data Availability

The datasets used and/or analysed during the current study are available from the corresponding author on reasonable request.
