# Supplementary material for: A frailty census of older adults in the emergency department and acute inpatient settings of a model 4 hospital in the Mid-West of Ireland
Source: Ir J Med Sci. 2024 Sep 19;193(6):3029–38. doi: 10.1007/s11845-024-03775-6 (PMC11666778; doi:10.1007/s11845-024-03775-6)
Supplement: Supplementary file 2 — Supplementary file2 (PDF 90 KB) [file 11845_2024_3775_MOESM2_ESM.pdf]

STROBE Statement—Checklist of items that should be included in reports of ***cross-sectional studies***

	Item No	Recommendation	Page No
**Title and abstract**	1	(*a*) Indicate the study's design with a commonly used term in the title or the abstract	Pg 1 Abstract
		(*b*) Provide in the abstract an informative and balanced summary of what was done and what was found	Pg 1 and 2 Abstract
**Introduction**			
Background/rationale	2	Explain the scientific background and rationale for the investigation being reported	Pg 2 and 3
Objectives	3	State specific objectives, including any prespecified hypotheses	Pg 2 and 3
**Methods**			
Study design	4	Present key elements of study design early in the paper	Pg 3 and 4
Setting	5	Describe the setting, locations, and relevant dates, including periods of recruitment, exposure, follow-up, and data collection	Pg 3 and 4 and Findings
Participants	6	(*a*) Give the eligibility criteria, and the sources and methods of selection of participants	Pg 3 and 4
Variables	7	Clearly define all outcomes, exposures, predictors, potential confounders, and effect modifiers. Give diagnostic criteria, if applicable	Pg 3 and 4
Data sources/ measurement	8*	For each variable of interest, give sources of data and details of methods of assessment (measurement). Describe comparability of assessment methods if there is more than one group	Pg 3, 4 and Findings
Bias	9	Describe any efforts to address potential sources of bias	Pg 3, 4, 12
Study size	10	Explain how the study size was arrived at	Pg 3 and 4
Quantitative variables	11	Explain how quantitative variables were handled in the analyses. If applicable, describe which groupings were chosen and why	Pg 3 and 4 and Findings
Statistical methods	12	(*a*) Describe all statistical methods, including those used to control for confounding	Pg 3 and 4 and Findings
		(*b*) Describe any methods used to examine subgroups and interactions	Pg 3 and 4 and Findings
		(*c*) Explain how missing data were addressed	Not required
		(*d*) If applicable, describe analytical methods taking account of sampling strategy	Pg 3 and 4 and Findings
		(*e*) Describe any sensitivity analyses	Crosstabulation for associations. P-values.
**Results**			
Participants	13*	(a) Report numbers of individuals at each stage of study—e.g. numbers potentially eligible, examined for eligibility, confirmed eligible, included in the study, completing follow-up, and analysed	Pg 4-9 for ED and Inpatient Cohort
		(b) Give reasons for non-participation at each stage	Not required

		(c) Consider use of a flow diagram	Not required
Descriptive data	14*	(a) Give characteristics of study participants (e.g. demographic, clinical, social) and information on exposures and potential confounders	Pg 3-9 for ED and Inpatients
		(b) Indicate number of participants with missing data for each variable of interest	Not required
Outcome data	15*	Report numbers of outcome events or summary measures	Pg 3 and 4.
Main results	16	(*a*) Give unadjusted estimates and, if applicable, confounder-adjusted estimates and their precision (e.g., 95% confidence interval). Make clear which confounders were adjusted for and why they were included	Pg 3-9
		(*b*) Report category boundaries when continuous variables were categorized	Pg 4-9
		(*c*) If relevant, consider translating estimates of relative risk into absolute risk for a meaningful time period	Not relevant
Other analyses	17	Report other analyses done—e.g. analyses of subgroups and interactions, and sensitivity analyses	Pg 4-9
**Discussion**			
Key results	18	Summarise key results with reference to study objectives	Pg 10, 11, 12
Limitations	19	Discuss limitations of the study, taking into account sources of potential bias or imprecision. Discuss both direction and magnitude of any potential bias	Pg 12 Para 2
Interpretation	20	Give a cautious overall interpretation of results considering objectives, limitations, multiplicity of analyses, results from similar studies, and other relevant evidence	Pg, 10, 11, 12, 13.
Generalisability	21	Discuss the generalisability (external validity) of the study results	Pg 12
**Other information**			
Funding	22	Give the source of funding and the role of the funders for the present study and, if applicable, for the original study on which the present article is based	Pg 13

*Give information separately for exposed and unexposed groups.

Note: An Explanation and Elaboration article discusses each checklist item and gives methodological background and published examples of transparent reporting. The STROBE checklist is best used in conjunction with this article (freely available on the Web sites of PLoS Medicine at http://www.plosmedicine.org/, Annals of Internal Medicine at http://www.annals.org/, and Epidemiology at http://www.epidem.com/). Information on the STROBE Initiative is available at www.strobe-statement.org.
