# Supplementary material for: A frailty census of older adults in the emergency department and acute inpatient settings of a model 4 hospital in the Mid-West of Ireland
Source: Ir J Med Sci. 2024 Sep 19;193(6):3029–38. doi: 10.1007/s11845-024-03775-6 (PMC11666778; doi:10.1007/s11845-024-03775-6)
Supplement: Supplementary file 3 — Supplementary file3 (PDF 479 KB) [file 11845_2024_3775_MOESM3_ESM.pdf]

Methods Data Analysis

Descriptive statistics were calculated for all included variables. This included frequency statistics for categorical variables and means (standard deviations) for scale variables. For each cohort – ED and Inpatients, chi-square tests of association were conducted to see if distributions of frailty and comorbidities significantly differed across age (<75, ≥75), gender (male, female), medical admission (yes vs. no), and surgical admission (yes vs. no). Frailty was investigated using validated clinical cut-offs on the CFS (not frail <5; frail ≥5). Comorbidities were investigated using the Charlson Comorbidity Index (mild 1-2; moderate 3-4; severe ≥5).

Inpatients

Demographics

Age

	N	%
<75	104	35.7%
>=75	187	64.3%

Gender

	N	%
Male	158	54.3%
Female	133	45.7%

Medical

	N	%
No	84	28.9%
Yes	207	71.1%

Surgical

	N	%
No	170	58.4%
Yes	121	41.6%

CFS_CAT

	N	%
Not Frail	46	15.8%
Frail	245	84.2%

Charlson_CAT

	N	%
Mild	68	23.4%
Moderate	132	45.4%
Severe	91	31.3%

CFS_SCORE

	N	%
2	4	1.4%
3	11	3.8%
4	31	10.7%
5	52	17.9%
6	94	32.3%
7	85	29.2%
8	14	4.8%

Descriptive Statistics

	N	Range	Minimum	Maximum	Mean	Std. Deviation
CFS_SCORE	291	6	2	8	5.83	1.275
Charlson_SCORE	291	11	1	12	4.04	2.342
Valid N (listwise)	291					

Crosstabulations CFS

CFS_CAT * Age Crosstabulation

			Age		
			<75	>=75	Total
CFS_CAT	Not Frail	Count	26	20	46
		Expected Count	16.4	29.6	46.0
		% of Total	8.9%	6.9%	15.8%
	Frail	Count	78	167	245
		Expected Count	87.6	157.4	245.0
		% of Total	26.8%	57.4%	84.2%
Total		Count	104	187	291
		Expected Count	104.0	187.0	291.0
		% of Total	35.7%	64.3%	100.0%

- Chi square test significant p=0.002. Those >= 75 more likely to be frail.

CFS_CAT * Medical Crosstabulation

			Medical		
			No	Yes	Total
CFS_CAT	Not Frail	Count	22	24	46
		Expected Count	13.3	32.7	46.0
		% of Total	7.6%	8.2%	15.8%
	Frail	Count	62	183	245
		Expected Count	70.7	174.3	245.0
		% of Total	21.3%	62.9%	84.2%
Total		Count	84	207	291
		Expected Count	84.0	207.0	291.0
		% of Total	28.9%	71.1%	100.0%

- Chi square test significant p = 0.004. Medical admission more likely to be frail.

CFS_CAT * Surgical Crosstabulation

			Surgical		
			No	Yes	Total
CFS_CAT	Not Frail	Count	18	28	46
		Expected Count	26.9	19.1	46.0
		% of Total	6.2%	9.6%	15.8%
	Frail	Count	152	93	245
		Expected Count	143.1	101.9	245.0
		% of Total	52.2%	32.0%	84.2%
Total		Count	170	121	291
		Expected Count	170.0	121.0	291.0
		% of Total	58.4%	41.6%	100.0%

- Chi-square test significant p=0.006, higher % of surgical admissions are not frail

CFS_CAT * Gender Crosstabulation

			Gender		
			Male	Female	Total
CFS_CAT	Not Frail	Count	30	16	46
		Expected Count	25.0	21.0	46.0
		% of Total	10.3%	5.5%	15.8%
	Frail	Count	128	117	245
		Expected Count	133.0	112.0	245.0
		% of Total	44.0%	40.2%	84.2%
Total		Count	158	133	291
		Expected Count	158.0	133.0	291.0
		% of Total	54.3%	45.7%	100.0%

- Chi-square not significant p = 0.144. No association between CFS Frailty and Gender.

Crosstabulations Charlson

Charlson_CAT * Age Crosstabulation

			Age		Total
			<75	>=75	
Charlson_CAT	Mild	Count	32	36	68
		Expected Count	24.3	43.7	68.0
		% of Total	11.0%	12.4%	23.4%
	Moderate	Count	48	84	132
		Expected Count	47.2	84.8	132.0
		% of Total	16.5%	28.9%	45.4%
	Severe	Count	24	67	91
		Expected Count	32.5	58.5	91.0
		% of Total	8.2%	23.0%	31.3%
Total		Count	104	187	291
		Expected Count	104.0	187.0	291.0
		% of Total	35.7%	64.3%	100.0%

- Chi-square significant p = 0.026, older age associated with more severe frailty

Charlson_CAT * Medical Crosstabulation

			Medical		Total
			No	Yes	
Charlson_CAT	Mild	Count	25	43	68
		Expected Count	19.6	48.4	68.0
		% of Total	8.6%	14.8%	23.4%
	Moderate	Count	33	99	132
		Expected Count	38.1	93.9	132.0
		% of Total	11.3%	34.0%	45.4%
	Severe	Count	26	65	91
		Expected Count	26.3	64.7	91.0
		% of Total	8.9%	22.3%	31.3%
Total		Count	84	207	291
		Expected Count	84.0	207.0	291.0

		% of Total	28.9%	71.1%	100.0%

- Chi-square non-significant p =0.227, no association between medical admission and severity of frailty.

Charlson_CAT * Surgical Crosstabulation

			Surgical		
			No	Yes	Total
Charlson_CAT	Mild	Count	39	29	68
		Expected Count	39.7	28.3	68.0
		% of Total	13.4%	10.0%	23.4%
	Moderate	Count	81	51	132
		Expected Count	77.1	54.9	132.0
		% of Total	27.8%	17.5%	45.4%
	Severe	Count	50	41	91
		Expected Count	53.2	37.8	91.0
		% of Total	17.2%	14.1%	31.3%
Total		Count	170	121	291
		Expected Count	170.0	121.0	291.0
		% of Total	58.4%	41.6%	100.0%

- Chi-square non-significant p =0.620, no association between surgical admission and severity of frailty.

Charlson_CAT * Gender Crosstabulation

			Gender		
			Male	Female	Total
Charlson_CAT	Mild	Count	34	34	68
		Expected Count	36.9	31.1	68.0
		% of Total	11.7%	11.7%	23.4%
	Moderate	Count	70	62	132
		Expected Count	71.7	60.3	132.0
		% of Total	24.1%	21.3%	45.4%
	Severe	Count	54	37	91

	Expected Count	49.4	41.6	91.0
	% of Total	18.6%	12.7%	31.3%
Total	Count	158	133	291
	Expected Count	158.0	133.0	291.0
	% of Total	54.3%	45.7%	100.0%

- Chi-square non-significant p =0.465, no association between gender and severity of frailty.

ED Cohort

Descriptives

Age Range

	N	%
<75	30	37.0%
>= 75	51	63.0%

Gender

	N	%
Male	35	43.2%
Female	46	56.8%

Med

	N	%
No	18	22.2%
Yes	63	77.8%

Surg

	N	%
No	53	65.4%
Yes	28	34.6%

CFS_CAT

	N	%
Not Frail	21	25.9%
Frail	60	74.1%

Charlson_CAT

	N	%
Mild	3	3.7%
Moderate	27	33.3%
Severe	51	63.0%

CFS_SCORE

	N	%
2	2	2.5%
3	6	7.4%
4	13	16.0%
5	15	18.5%
6	20	24.7%
7	20	24.7%
8	5	6.2%

Descriptive Statistics

	N	Range	Minimum	Maximum	Mean	Std. Deviation
CFS_SCORE	81	6	2	8	5.54	1.484
Charlson_SCORE	81	12	1	13	5.75	2.672
Valid N (listwise)	81					

Crosstabs CFS

CFS_CAT * Age Range Crosstabulation

			Age Range		
			<75	>= 75	Total
CFS_CAT	Not Frail	Count	11	10	21
		Expected Count	7.8	13.2	21.0
		% of Total	13.6%	12.3%	25.9%
	Frail	Count	19	41	60
		Expected Count	22.2	37.8	60.0
		% of Total	23.5%	50.6%	74.1%
Total		Count	30	51	81
		Expected Count	30.0	51.0	81.0
		% of Total	37.0%	63.0%	100.0%

- Chi-square not significant p=0.153. No association between age and frailty

CFS_CAT * Med Crosstabulation

			Med		
			No	Yes	Total
CFS_CAT	Not Frail	Count	8	13	21
		Expected Count	4.7	16.3	21.0
		% of Total	9.9%	16.0%	25.9%
	Frail	Count	10	50	60
		Expected Count	13.3	46.7	60.0
		% of Total	12.3%	61.7%	74.1%
Total		Count	18	63	81
		Expected Count	18.0	63.0	81.0
		% of Total	22.2%	77.8%	100.0%

- Chi-square not significant p = 0.084, no association between medical admissions and frailty.

CFS_CAT * Surg Crosstabulation

			Surg		
			No	Yes	Total
CFS_CAT	Not Frail	Count	11	10	21
		Expected Count	13.7	7.3	21.0
		% of Total	13.6%	12.3%	25.9%
	Frail	Count	42	18	60
		Expected Count	39.3	20.7	60.0
		% of Total	51.9%	22.2%	74.1%
Total		Count	53	28	81
		Expected Count	53.0	28.0	81.0
		% of Total	65.4%	34.6%	100.0%

- Chi-square not significant p = 0.232, no association between surgical admissions and frailty.

CFS_CAT * Gender Crosstabulation

			Gender		Total
			Male	Female	
CFS_CAT	Not Frail	Count	11	10	21
		Expected Count	9.1	11.9	21.0
		% of Total	13.6%	12.3%	25.9%
	Frail	Count	24	36	60
		Expected Count	25.9	34.1	60.0
		% of Total	29.6%	44.4%	74.1%
Total		Count	35	46	81
		Expected Count	35.0	46.0	81.0
		% of Total	43.2%	56.8%	100.0%

- Chi-square not significant p = 0.465, no association between gender and frailty.

Crosstabulation Charlson Comorbidity Index (CCI)

Crosstabulations for the CCI were run on the moderate and severe groups only due to the mild group having insufficient counts to be included in the analysis.

Charlson_CAT * Age Range Crosstabulation

			Age Range		Total
			<75	>= 75	
Charlson_CAT	Moderate	Count	12	15	27
		Expected Count	9.7	17.3	27.0
		% of Total	15.4%	19.2%	34.6%
	Severe	Count	16	35	51
		Expected Count	18.3	32.7	51.0
		% of Total	20.5%	44.9%	65.4%
Total		Count	28	50	78
		Expected Count	28.0	50.0	78.0
		% of Total	35.9%	64.1%	100.0%

- Chi-square non-significant ($p = 0.252$), no association between CCI and age range.

CCI_CAT * Med Crosstabulation

Charlson_CAT * Med Crosstabulation

			Med		Total
			No	Yes	
Charlson_CAT	Moderate	Count	8	19	27
		Expected Count	5.5	21.5	27.0
		% of Total	10.3%	24.4%	34.6%
	Severe	Count	8	43	51
		Expected Count	10.5	40.5	51.0
		% of Total	10.3%	55.1%	65.4%
Total		Count	16	62	78
		Expected Count	16.0	62.0	78.0
		% of Total	20.5%	79.5%	100.0%

- Chi-square non-significant ($p = 0.147$) no association between CCI and medications.

CCI_CAT * Surg Crosstabulation

Charlson_CAT * Surg Crosstabulation

			Surg		
			No	Yes	Total
Charlson_CAT	Moderate	Count	17	10	27
		Expected Count	18.0	9.0	27.0
		% of Total	21.8%	12.8%	34.6%
	Severe	Count	35	16	51
		Expected Count	34.0	17.0	51.0
		% of Total	44.9%	20.5%	65.4%
Total		Count	52	26	78
		Expected Count	52.0	26.0	78.0
		% of Total	66.7%	33.3%	100.0%

- Chi-square non-significant (p=0.614), no association between CCI and surgery

CCI_CAT * Gender Crosstabulation

Charlson_CAT * Gender Crosstabulation

			Gender		
			Male	Female	Total
Charlson_CAT	Moderate	Count	10	17	27
		Expected Count	11.8	15.2	27.0
		% of Total	12.8%	21.8%	34.6%
	Severe	Count	24	27	51
		Expected Count	22.2	28.8	51.0
		% of Total	30.8%	34.6%	65.4%
Total		Count	34	44	78
		Expected Count	34.0	44.0	78.0
		% of Total	43.6%	56.4%	100.0%

- Chi-square non-significant (p=0.396), no association between CCI and gender.
